# Navigating the 2021 ACPSEM ROMP workforce model: insights from a single institution

**DOI:** 10.1007/s13246-024-01406-z

**Published:** 2024-02-29

**Authors:** Broderick Ivan McCallum-Hee, Godfrey Mukwada

**Affiliations:** 1 Department of Radiation Oncology, Sir Charles Gairdner Hospital, 6009 Nedlands, WA Australia; 2 School of Physics, Mathematics and Computing, The University of Western Australia, 6009 Crawley, WA Australia

**Keywords:** Staffing, Medical physics, Radiation Oncology, ROMP, Workforce, ACPSEM

## Abstract

Workforce modelling for Radiation Oncology Medical Physicists (ROMPs) is evolving and challenging, prompting the development of the 2021 Australasian College of Physical Scientists and Engineers in Medicine (ACPSEM) ROMP Workforce (ARW) Model. In the exploration of this model at Sir Charles Gairdner Hospital, a comprehensive productivity exercise was conducted to obtain a detailed breakdown of ROMP time at a granular level. The results provide valuable insights into ROMP activities and enabled an evaluation of ARW Model calculations. The findings also capture the changing ROMP role as evidenced by an increasing involvement in consultation and advisory tasks with other professionals in the field. They also suggest that CyberKnife QA time requirements in the data utilised by the model may need to be revised. This study emphasises features inherent in the model, that need to be understood if the model is to be applied correctly.

## Introduction

Radiation Oncology Medical Physicists (ROMPs) are an essential, highly specialised workforce in Radiation Oncology. Accurately modelling the required number of ROMPs to meet the clinical needs of a specific centre is critical for ensuring safe and effective patient care. However, this task is complex due to the rapidly evolving nature of ROMP role in the evolving field of radiation therapy. Several authors have attempted to develop models to calculate ROMP workforce requirements at international and regional levels [1–7]. The general approach involves scaling a full-time equivalent (FTE) value by the number of machines, patients or treatment courses, and other activities.

In 2001, the Australasian College of Physical Scientists and Engineers in Medicine (ACPSEM) endorsed the Formula 2000 model [2] for centres in Australia and New Zealand. However, as new techniques became routine and novel approaches emerged, the applicability of this model to modern centres diminished. To address this, the ACPSEM released the activity-based 2021 ACPSEM ROMP Workforce (ARW) Model [1], which was developed using the IAEA model [4] and a regional survey of radiation oncology centres.

In 2012, the ROMP staffing levels at Sir Charles Gairdner Hospital (SCGH) in Western Australia were determined based on the Formula 2000 model. The department had 5 Varian Clinac linear accelerators (linacs), an intraoperative x-ray unit, a high dose rate (HDR) brachytherapy unit and 4 treatment planning systems (TPSs). The treatment techniques employed included 3D conformal, intensity modulated and stereotactic radiation therapy, stereotactic radiosurgery, as well as low dose rate (LDR) and HDR brachytherapy. The department was and still is standalone and part of a public teaching hospital. Currently, SCGH is equipped with 5 Varian TrueBeam linacs (3 with 6-DoF couches), a CyberKnife, a HDR brachytherapy unit and 2 TPSs (Eclipse and Precision). Although the overall patient mix remains similar, treatment modalities have shifted. IORT and LDR brachytherapy are no longer clinically used, with these patients now being treated with EBRT and CyberKnife respectively. The Formula 2000 model indicated that SCGH should have 14 FTE physicists and 2 FTE physicist registrars. However, since then, the composition of the ROMP team has changed, and SCGH now has 12.1 FTE physicists and 4 FTE registrars. Compared to 2012, over 87% of linac patients are treated using volumetric modulated arc therapy (VMAT) inclusive of stereotactic body radiation therapy (SBRT) and 16% of all patients are treated using the CyberKnife. More advanced techniques require more patient-specific QA (including plan checking) and require more physics consultation during the planning process. SCGH’s patient-specific QA includes EPID-based QA for linac VMAT and film for SBRT/CyberKnife.

In 2022 and 2023, SCGH conducted a comprehensive productivity exercise to delve into the specifics of ROMP work activities and gain insights into time allocation patterns. The primary objective was to develop an informed understanding of local work practices, enabling a valid comparison with the ARW Model. The purpose of this paper is to present the results obtained from the productivity exercise, analyse the implications for ARW Model calculations, and contribute to the broader discussion on optimising ROMP workforce planning.

## Methods

### Productivity exercise

The ARW Model provides a ROMP activity time breakdown based on averages across surveyed radiation oncology departments in Australia and New Zealand. However, to ensure accurate calculations for SCGH, a daily productivity exercise to obtain data specific to the institution was conducted over five months in two periods August to November 2022 and March 2023. To capture data a survey was developed using Microsoft Forms, an online form-creation tool. Work activities were categorised into nine groups: Linac, CyberKnife, CT, Brachytherapy, Education & Training, Commissioning, Admin & Meetings, Project Work, and Other. Participants recorded the number of hours spent on each activity, and when logging hours under the ‘Other’ category, were required to provide a brief description. The survey was designed to align with SCGH work practices and facilitate a high completion rate. The activity breakdown obtained was then mapped onto the corresponding activities used in the ARW Model. A comprehensive overview of the activities captured in the productivity exercise is included in Table 1.

To support the exercise, a reminder system was implemented using Microsoft Power Automate and Microsoft Lists. A ‘Participant’ list contained the email addresses of all participants and a ‘Reminder Required’ list tracked participants who had not submitted the survey on a given day. Every workday at 4 PM during the exercise period a reminder email was sent by Power Automate. Participants who had not submitted the survey for multiple days were also followed up via email or in person using a simple Microsoft Excel sheet linked to the survey.

### Case volume, standard work hours, and equipment data collection

Information from the SCGH Elekta MOSAIQ radiation oncology information system provided data on case numbers. The past year’s patient appointment and quality checklist tasks were extracted using Elekta’s Crystal Reports tool. Appointment data included the number of CT scans, treatment visits and immobilisation device fitting sessions. Quality checklist data captured all other department tasks recorded in MOSAIQ. Cross-checking the count of selected physics-specific QA activities from MOSAIQ with a separate plan quality metric trend log (maintains historic plan gamma pass rates, physicist comments, etc.) enabled data verification.

ROMP standard work conditions, such as daily hours and annual leave, were obtained from the ‘WA Health System - HSUWA - Pacts Industrial Agreement 2022’ [8] which covers medical physicists in Western Australia. All equipment or software in the department requiring ROMP time was counted. Physics time per patient case was determined by surveying physicists in the department.

## Results

### Productivity exercise

The productivity exercise occurred from August to November 2022 and in March 2023. The staff completion rate for the first period was approximately 50%, while for the second period, it reached 100%. Manual follow-ups on incomplete responses and use of the reminder system only occurred in the March period. To enhance the accuracy and representativeness of the combined results, the activity breakdown from each period was weighted by its corresponding response rate (0.5 for the first period and 1 for the second).

**Fig. 1:**
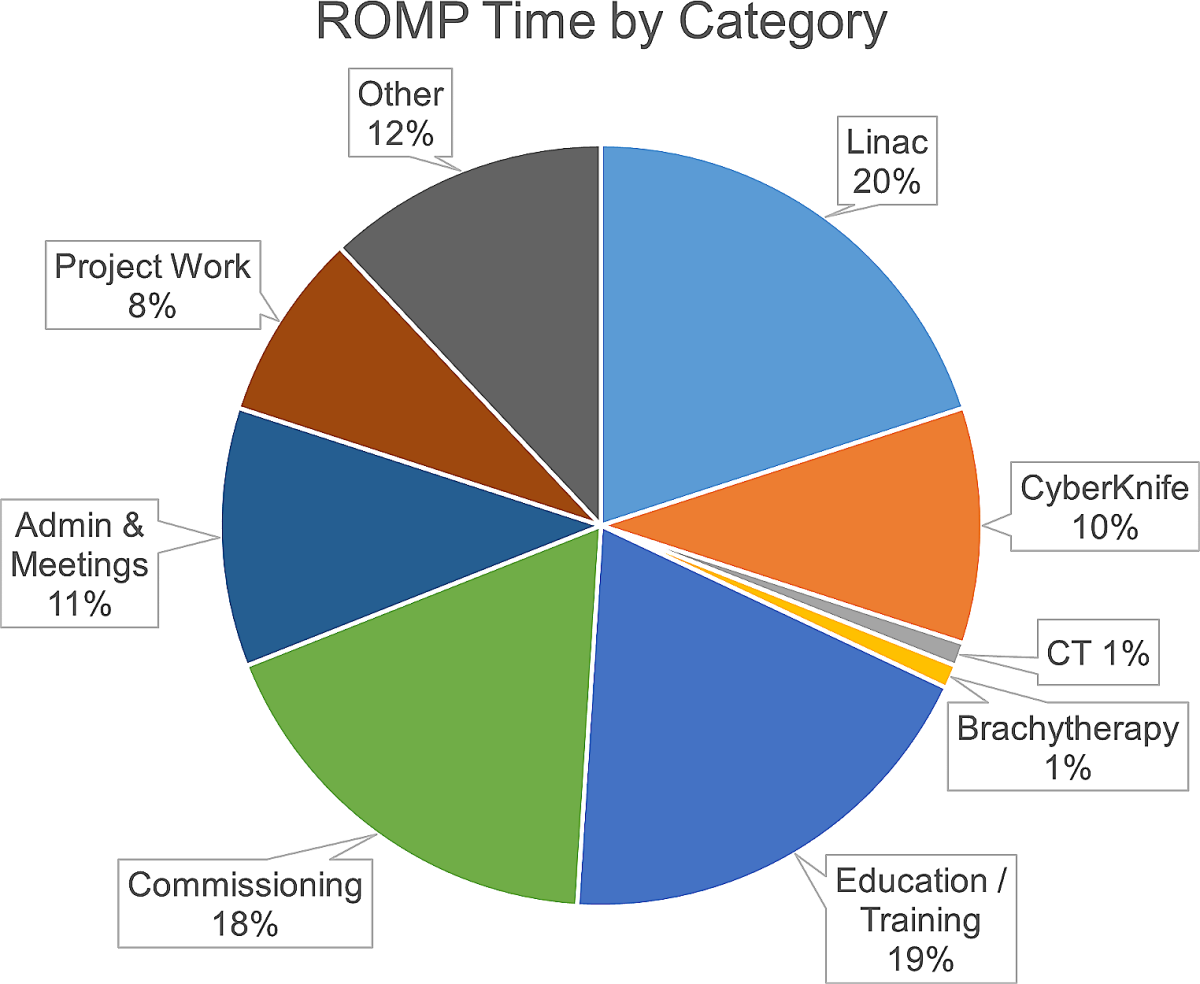
ROMP time broken into categories. Data for individual activities contained within each category is provided in Table 1

Figure 1 gives a breakdown of survey responses by category, and Table 1 presents the percentages of time spent on specific activities. Table 2 contains the results from mapping activities to their corresponding categories used in the ARW Model and the estimate from the model. To map time from the ‘Other’ category in the productivity exercise the descriptions provided with individual entries were checked.

Using the annual ROMP work hours, percentage of time allocated to specific activities, and number of machines at SCGH, it is possible to estimate the ROMP QA minutes per unit per year. Table 3 shows these values, along with the average minutes used by the ARW Model and a ratio between the two. In the calculation, the ‘linac’ category included the sum of ‘Linac’, ‘CBCT’, ‘OBI’, and ‘EPID’, while the ‘CyberKnife’ category included the sum of ‘CyberKnife’ and ‘Non orthogonal kV’.

**Table 1:** Percentage of ROMP time spent in each category and specific activity captured in the daily survey conducted as a part of the SCGH productivity exercise

Category	Specific Activity	Percentage of Time (%)
Linac (inc. Treatment Planning)	Patient specific QA − 1st check	5.0
	Patient specific QA − 2nd check	4.9
	Scheduled machine QA	5.0
	Unscheduled machine QA	1.5
	Advising RTs/ROs	3.4
CyberKnife (inc. Treatment Planning)	Patient specific QA − 1st check	2.9
	Patient specific QA − 2nd check	0.6
	Scheduled machine QA	4.7
	Unscheduled machine QA	0.9
	Advising RTs/ROs	0.7
CT	4DCT	1.2
	Scheduled QA	0.2
	Unscheduled machine QA	0.1
Brachytherapy	Plan Checks	0.0
	Morning QA	0.2
	Treatment	0.3
	Source Exchange	0.3
Education / Training	TEAP work	14.5
	CPD related work	2.6
	Supervising research	2.2
Commissioning	Treatment Equipment	11.4
	Dosimetry Equipment	1.4
	Auxiliary Equipment	0.1
	Treatment Techniques	4.6
	Software	0.4
Admin & Meetings	Meetings	7.3
	Scheduling / Admin	3.8
Project Work	Project Work	8.4
Other	Updating SOPs / Spreadsheets	2.5
	IT Work	1.9
	Radiation Safety	0.6
	Dosimetry QA	0.3
	Patient Data Retrievals	0.5
	Other	6.5

**Table 2:** Percentage of SCGH ROMP time spent in the categories and activities used by the ARW Model. Produced through mapping productivity exercise data to appropriate corresponding activities

	Category	Specific Activity	SCGH Survey Data			ARW AUS/NZ Average		
Non-Patient or Equipment QA	Education	Classroom and departmental teaching/tutorials	0.5%	**15%**	**68.6%**	2.8%	**9.1%**	**68.7%**
		TEAP activities	14.5%			6.4%		
	Quality and Safety	Radiation safety and protection	0.6%	**5.2%**		4.1%	**19.6%**	
		Follow-up evaluations - PATIENT	0.5%			4.5%		
		Follow-up evaluations - SYSTEM				5.4%		
		Indirect patient care	3.6%			2.8%		
		Internal / External auditing and accreditation	0.5%			2.8%		
	Clinical and service	Clinical development	8.4%	**26.3%**		9.9%	**20.3%**	
		Service development	17.9%			10.4%		
	Other Professional activities	Research	2.2%	**22.1%**		3.1%	**19.7%**	
		Continuing professional development	2.6%			3.4%		
		Administration-management	9.3%			6.5%		
		Document management	2.5%			4.2%		
		College activities	1.0%			1.6%		
		Other	4.5%			1.0%		
Patient or Equipment QA			**31.4%**			**31.3%**		

**Table 3:** SCGH and ARW model ROMP QA minutes per unit per year and the ratio between them

Equipment	SCGH Minutes	ARW Model Minutes	Ratio (SCGH / ARW)
Linac	20,797	14,968	1.4
CyberKnife	90,282	23,430	3.9
CT Sim	4,353	3,151	1.4
Brachy HDR / PDR	8,706	6,142	1.4

### ARW model ROMP FTE estimate

Table 4 presents a breakdown of SCGH case load with used ARW Model ‘physics time per case’ and Table 5 the equipment volumes.

Inputting data into the ARW Model yields an estimated qualified ROMP FTE requirement of 12.96 for SCGH. This estimate is 0.86 higher than the qualified number of staff at SCGH, as there are 12.1 FTE physicists and 4 FTE physicist registrars.

**Table 4:** SCGH case load inputs for the ARW Model. Note, ARW items not included either had 0 case load or negligible physics time per case

Activity	Volume of cases	Physics time per case
EBRT (Simulation, Planning, QA measurement, QA analysis, Treatment delivery and IVD)		
3DCRT	160	Mid
VMAT/IMRT/Tomotherapy	1800	High
Electrons	110	Mid
SABR simple	75	High
SABR complex	10	High
Adaptive RT	1	High
Cyberknife	413	High
TBI	1	High
EBRT: additional activities		
Motion Mgmt	434	High
Block cutting/accessories/output factor measurement/bolus	319	Mid
Advice / measurements for implanted devices	17	Mid
Evaluation/advice during treatment	384	High
Brachytherapy		
Simple insertion of applicator or mould placement without image guidance	11	High

**Table 5:** SCGH equipment inputs for the ARW Model

Equipment	Total Count
Linac	5
Cyberknife	1
CT Sim	1
Brachy HDR / PDR	1
CBCT	5
OBI	5
Non orthogonal KV	1
EPID	5
2D, 3D and 4D TPS per DB	2
R&V network/OIS	1
Image processing and registration systems	2
Independent dose verification systems	2
Absolute dosimetry equipment	13
Relative dosimetry equipment	10
Survey and monitoring equipment	8
Workshop	1
SRT / SBRT / SRS / IORT equipment	1

## Discussion

The utilisation of an electronic reminder system and manual follow-ups on incomplete responses resulted in a doubled survey completion rate. This emphasises the significance of reminders and follow-ups in achieving a high response rate.

When comparing activity breakdown results from SCGH with averages from Australia/New Zealand used in the ARW Model, significant variations were observed in individual categories and activities. However, the consistency in ROMP time to patient and equipment QA is noteworthy. Despite wide ranges in the underlying data of the ARW averages, the difference is only 0.1%. The variation in individual categories may result from differences in survey design and activity category attribution. The comparative overweighting of the ‘Education’ category may result from SCGH’s relatively large registrar cohort with a qualified staff to registrar ratio of 3:1. The Formula 2000 model recommended a ratio of 5:1, with an expectation of it eventually reducing to 10:1. In 2021, the ACPSEM ROMP database recorded a ratio of 4:1 across Australia and New Zealand. Additionally, the exercise did not cover a whole year so activity weightings may be biased towards work conducted during that time. For example, there were no radiation incidents, shielding calculations or surveying conducted during the exercise. By contrast, other non-routine work was captured such as radiation safety work due to an increase in VMAT/SBRT cases and the installation of a new CyberKnife head during the exercise.

The estimated annual machine QA minutes for all equipment at SCGH is significantly higher than the Australia/New Zealand averages provided in the ARW Model. The ‘Linac’, ‘CT Sim’, and ‘Brachy’ categories were 1.4 times higher, while the ‘CyberKnife’ category was four times higher.

The ARW Model’s time requirements for CyberKnife QA may need revision, given SCGH data constitutes half of the Australian dataset. The productivity exercise suggests that SCGH’s initial estimates in the facility survey, utilised by the ARW model, were underestimates. During the survey period, CyberKnife underwent its annual QA and encountered a series of breakdowns resulting in increased recorded ROMP times. To manage this time was diverted from quality improvement activities and overtime recorded. It is worth noting that CyberKnife breakdowns are not unusual as SCGH encounters them semi-regularly however their duration can significantly vary. Robotic radiosurgery systems, like CyberKnife, are inherently complex and require thorough QA [9]. Generally, CyberKnife repair and maintenance work results in repeated physics QA as fine tuning is iterative. This repetition makes it difficult to predict the exact physics QA time required. Additionally, some of the recorded time may reflect physicists participating in activities as part of their CyberKnife training which was not considered in initial facility survey estimates. Given the unpredictable nature of CyberKnife breakdowns it is difficult to accurately determine annual QA time requirements and would require monitoring over a longer period.

Accounting for machine-specific breakdown behaviours is currently beyond the scope of workforce modelling. Although no specific major breakdown events occurred for the linacs, CT or brachytherapy machines as for the CyberKnife, the impact of breakdowns increased the machine QA time requirement captured by the productivity exercise. Future models could provide finer granularity to enable such consideration through inputting factors such as machine age and centre-specific circumstances. The ARW Model accommodates a range of physics time per case for patient related activities and a similar approach could be applied to QA time.

The ARW Model while applicable to all centres due to its activity-based approach is constructed from data biased towards current private centre staffing levels. A previous survey found that private centres maintain lower staff levels than public centres across various radiation oncology professions, including ROMPs [10]. It found public centres had an average of 1.6 ROMPs per linac, while private centres had only one. The ARW Model is based on data from 54 private, 37 public, and 7 public-private centres (62% private or public-private). As a rough estimate, if all public and public-private centres engage 1.6 times the number of ROMPs compared to private centres, this gives a weighted average factor of 1.3 across survey data. Therefore, the model’s FTE estimates are likely lower than current staff levels at public centres and higher than at private centres. Notably, this factor is comparable in magnitude to the difference between the annual machine QA time indicated by results of the SCGH productivity exercise and that used in the ARW Model.

In the ARW Model data, 82% of the centres were networked [1]. The ARW report notes that networked departments exhibit similar workforce practices with some sharing ROMP resources [1]. This arrangement grants networked departments greater flexibility to manage varying workloads than standalone centres. As a result, networked departments may be able to function with fewer ROMPs, handling higher workload periods through utilisation of pooled resources. Considering the substantial proportion of the model’s data sourced from networked departments, the accuracy of predictions for standalone departments such as SCGH may be reduced.

During ARW model data collection no obvious commonality in workforce practice and time spent on activities was found, particularly for non-networked centres [1]. When applying the model local workforce practices should therefore be considered as it is difficult to compare departments. It is also important to consider that this exercise is only one centre’s experience. For example, SCGH has a practice of scheduling two physicists per machine QA which may cause the ARW Model to underestimate FTE requirements. In-depth discussion towards the merits and demerits of two-person versus one-person machine QA is beyond the scope of this study. Past incidents in radiation oncology suggest relying solely on a single physicist’s work can be hazardous; however, the implementation of double-checking should be carefully considered [11, 12]. Comparing SCGH with the ARW Model indicates an opportunity for reflecting on work practices at SCGH which could reduce observed differences by enhancing efficiency and optimisation. For example, a planned transition from film to SRS MapCheck for patient-specific QA is expected to increase efficiency. Due to complex treatments and commissioning of new techniques, SCGH physicists are also heavily involved in difficult cases and multidisciplinary team meetings.

The current number of qualified ROMPs at SCGH is lower than the ARW Model estimate. However, the overall number of physicists is higher, indicating that registrars may support a slight deficit of qualified staff. The ARW Model allows facilities to determine the contribution of registrars and given the current ACPSEM ROMP training entrustment model it is expected they will cover some portion of clinical work. Considering the model is not designed to define a standard of what is considered best practice and all discussed factors the current ROMP staff levels at SCGH were considered reasonable.

A significant 4.1% of ROMP time was spent providing advice to radiation therapists and oncologists (3.4% related to linac and 0.7% to CyberKnife). Providing advice has always been a part of the ROMP role. However, the increasing complexity of techniques and case volume necessitates a growing need for ROMP FTE in this area. The ARW Model’s volume-based activity approach can effectively account for this, provided time per case requirements evolve in tandem with the practice. The field of medical physics, as discussed by other authors, is evolving [13–16]. The introduction of faster QA, more reliable machines, and automation is expected to reduce the time spent on many existing activities. This shift will require ROMPs to engage in emerging areas such as therapy personalisation, artificial intelligence, and complex simulation. For instance, at SCGH, automation has significantly reduced the time required to review basic plan parameters, leaving more time for consulting on challenging cases and clinical development. In the future, it is anticipated ROMPs will have increased involvement in direct patient care [17]. While the ARW Model provides valuable guidance on current ROMP requirements, it is important to remain mindful of the changing landscape of the field and potential variations that may arise in the future.

## Conclusion

The ROMP staffing level at SCGH has varied over the years, and recently a comprehensive productivity exercise exploring local work practices and time allocation patterns was conducted. Through this exercise a detailed activity breakdown of ROMP time was produced and compared to the ARW Model. The breakdown generally agrees with data collected in Australia and New Zealand during development of the ARW Model, despite some variation for specific activities / categories. SCGH had a comparative overweighting of the ‘Education’ category attributable to the relatively large registrar cohort. The breakdown also highlights the evolving role of ROMPS, as evidenced by an increasing involvement in general consultation and advisory tasks, reflecting the evolving nature of the field of medical physics. SCGH machine QA time requirements for all equipment were 1.4 times higher than those in the ARW Model except for CyberKnife which was four times higher. Since a large proportion of the ARW Model’s CyberKnife data comes from SCGH, the QA time requirements may need revision. But more data is necessary to determine accurate QA time requirements because CyberKnife breakdowns are hard to predict. In accounting for observed deviations the potential bias of the ARW Model towards private/public-private and networked centres which have on average fewer ROMPs and account for 62% and 82% of the underlying data respectively was explored. Networked centres may be able to function with fewer ROMPs by leveraging combined resources. The use of efficient practices, equipment, and systems will also impact ROMP requirements. The ARW Model uses an activity-based approach ensuring it is still applicable to other centres, however, when applying the model planners should also carefully consider local workforce practices. As the field of medical physics evolves workforce models will need to be updated to ensure their outputs remain accurate.
